# Protocol: Mapping social networks, social influence and sexual health among youth in rural KwaZulu-Natal, the Sixhumene cohort study

**DOI:** 10.12688/wellcomeopenres.17896.1

**Published:** 2022-05-26

**Authors:** Vuyiswa Nxumalo, Siyabonga Nxumalo, Theresa Smit, Thandeka Khoza, Fikile Mdaba, Thulile Khumalo, Beniamino Cislaghi, Nuala McGrath, Janet Seeley, Maryam Shahmanesh, Guy Harling

**Affiliations:** 1 Africa Health Research Institute, KwaZulu-Natal, South Africa; 2 Department of Global Health and Development, London School of Hygiene & Tropical Medicine, London, WC1H 9SH, UK; 3 Faculty of Medicine, University of Southampton, Southampton, SO16 6YD, UK; 4 Faculty of Social Sciences, University of Southampton, Southampton, SO17 1BJ, UK; 5 Institute for Global Health, University College London, London, WC1E 6JB, UK; 6 School of Nursing and Public Health, University of KwaZulu Natal, Durban, 4041, South Africa; 7 MRC/Wits-Agincourt Unit, University of the Witwatersrand, Johannesburg, Gauteng, 2193, South Africa; 8 Department of Epidemiology, Harvard T.H. Chan School of Public Health, Boston, MA, 02115, USA; 9 Harvard Center for Population and Development Studies, Harvard Univeristy, Cambridge, MA, 02138, USA

**Keywords:** South Africa, sexual health, HIV, HSV-2, social support, social influence

## Abstract

**Background**: Sexual behaviour and sexually transmitted infections are strongly affected by social connections, and interventions are often adapted more readily when diffused through social networks. However, evidence on how young people acquire ideas and change behaviour through the influence of important social contacts is not well understood in high-HIV-prevalence settings, with the result that past peer-led HIV-prevention interventions have had limited success.

**Methods**: We therefore designed a cohort study (named Sixhumene or ‘we are connected’) to follow young people in three rural and small-town communities in uMkhanyakude district, KwaZulu-Natal, South Africa, and the people that these youth identify as important in their lives. We will interview them five times over three years, at each visit collecting information on their socioeconomic, social and sexual health lives, and testing them for HIV and herpes simplex virus 2 (HSV-2). We will use this information to understand how these young people’s sexual health decisions are formed. This will include evaluating how poor sexual health outcomes are correlated across social networks, how youth mimic the attitudes and behaviours of those around them, who is at greatest risk of acquiring HIV and HSV-2, and who might be most influential within communities and thus best able to promote protective interventions.

**Discussion**: The information gathered through this study will allow us to describe social connection and influence spread through these real-world social networks, and how this leads to sexual health outcomes. Sixhumene will provide vital inputs for mathematical models of communities and spreading processes, as well as inform the development of effective interventions to protect the sexual health of community members through appropriate targeting with optimised messaging requiring fewer resources.

## Introduction

HIV prevalence and incidence among young South Africans remains very high ( Simbayi *et al*., 2019; Vandormael *et al*., 2019), despite the roll-out of several efficacious interventions (e.g., antiretroviral therapy ( Bor *et al*., 2013), voluntary medical male circumcision ( Davis *et al*., 2019; World Health Organization, 2017)). Continuing HIV transmission is in part due to non-uptake of prevention, treatment and care opportunities ( Baisley *et al*., 2018; Chimbindi *et al*., 2018; Haber *et al*., 2017; Iwuji *et al*., 2016; Lamb *et al*., 2014). Risk behaviours, and intervention uptake behaviours, are not randomly spread across populations, but tend to cluster in self-reinforcing high/low risk cliques due in part to social norms ( Kohler *et al*., 2007; Mulawa *et al*., 2016; Perkins & Berkowitz, 1986; Yamanis *et al*., 2017). Reaching at-risk cliques and changing their social norms is thus likely to be central to improving intervention uptake and HIV outcomes.

Uptake of many interventions has been improved through delivery by peers or influential others ( Latkin *et al*., 2013; Valente, 2012). Such an approach impacts social norms within high-risk cliques by leveraging stable ties of trust and respect: stable social ties are particularly important for behaviour change that requires multiple exposures to an idea before it is internalized, i.e. complex contagion ( Centola & Macy, 2007). Key social ties for young people typically comprise peers and family. Peer-led sexual health interventions have been used extensively ( Simoni *et al*., 2011), although few have explicitly considered social networks ( Wang *et al*., 2011). Sexual health interventions led by influential community members have been effective in US high-risk communities ( Kelly *et al*., 1991; Miller *et al*., 1998; Sikkema *et al*., 2000). Influential students have successfully supported non-smoking in the United Kingdom ( Campbell *et al*., 2008) and changed bullying social norms in the United States ( Paluck & Shepherd, 2012).

Early efforts to implement peer-led interventions in sub-Saharan African settings had limited impact on biological outcomes ( Medley *et al*., 2009), perhaps due to not enrolling truly influential peers as intervention leaders ( Fritz *et al*., 2011; Mason-Jones *et al*., 2011). More recent work has highlighted the role of socially close peers in behaviours such as HIV testing and initiation of antiretroviral therapy ( Brown *et al*., 2020; Conserve *et al*., 2019; Fearon *et al*., 2019; Yamanis *et al*., 2017). While evidence suggests that parental or other family-led communication efforts relating to sexual and reproductive health face barriers due to social taboos relating to sexual discussion and limited parental knowledge ( Armistead *et al*., 2014; Coetzee *et al*., 2014; Phetla *et al*., 2008), a comprehensive view of the relative impacts of peer and other social connections on health behaviours and outcomes among youth would assist in the development of interventions built around social networks ( Harling & Tsai, 2019).

We will conduct a network-structured cohort study to understand how behaviours cluster within social networks, in a rural KwaZulu-Natal setting with very high HIV prevalence and ongoing transmission. The study will focus on the late adolescence and early adulthood period (16–27 years). These are the ages at which local respondents first report sexual activity and pregnancy ( McGrath *et al*., 2009; Moultrie & McGrath, 2007), and also peak age for HIV acquisition and when other sexually transmitted infections are highly prevalent ( Akullian *et al*., 2021; Francis *et al*., 2018). We named the study *Sixhumene*, or “we are connected” in isiZulu, the primary local language, to reflect our core aim – to understand what processes generate and motivate sexual and reproductive health among youth and thus to determine how social processes affect HIV acquisition and linkage to care, and self-reported sexual health behaviours.

## Protocol

The current protocol is version 1.4 dated 24 January 2022.

### Study aims

The overall goal of the Sixhumene study is to determine how social processes are associated with sexual behaviours and thus risk for acquiring and transmitting HIV and herpes simplex virus 2 (HSV-2) among young people in rural KwaZulu-Natal, South Africa. We plan to achieve this goal through four objectives. Our primary objective is to understand how poor sexual health outcomes are correlated across social networks hypothesizing that (i) HSV-2 and HIV incidence, and (ii) non-linkage to HIV care, are higher for those with:

a) More social contacts living with HIV and HSV-2, and more contacts living with HIV but not linked to care, respectively; and
b) More peer social contacts.

We also have three secondary objectives. First, we aim to determine how social processes are associated with self-reported risk behaviours, hypothesizing that over time young people:

a) Change their behaviours to mirror those of their social contacts; and
b) Form friendships preferentially with those reporting similar behaviours.

Second, we aim to identify which young people are at greatest risk of acquiring HIV and HSV-2 based on their own characteristics, those of their social contacts and those of their communities. Finally, we aim to identify which young people are most influential on their peers’ risk behaviours and HIV and HSV-2 acquisition risk, by analysing changing outcomes within respondents and their social networks.

### Study setting

Sixhumene will be conducted within the Africa Health Research Institute’s Population Intervention Platform (AHRI PIP) in Hlabisa subdistrict, uMkhanyakude district, KwaZulu-Natal province, South Africa ( Gareta *et al*., 2021). AHRI has been conducting annual interviews in all PIP households for over 15 years, capturing socio-demographic, health behaviour, health status and HIV testing information. The area covers ~850km ^2^ and ~150,000 individuals who are members of over 20,000 households; while largely rural, the PIP includes the town of KwaMsane, with ~30,000 residents. UMkhanyakude district is ranked among the lowest socioeconomic areas in the country ( Fransman & Yu, 2019). HIV seropositivity is very common in the area, with ~20% of men and ~40% of women aged 15–54 living with HIV ( Vandormael *et al*., 2019). The working languages of the AHRI PIP surveillance area are isiZulu and English.

### Study design

**Figure 1.**
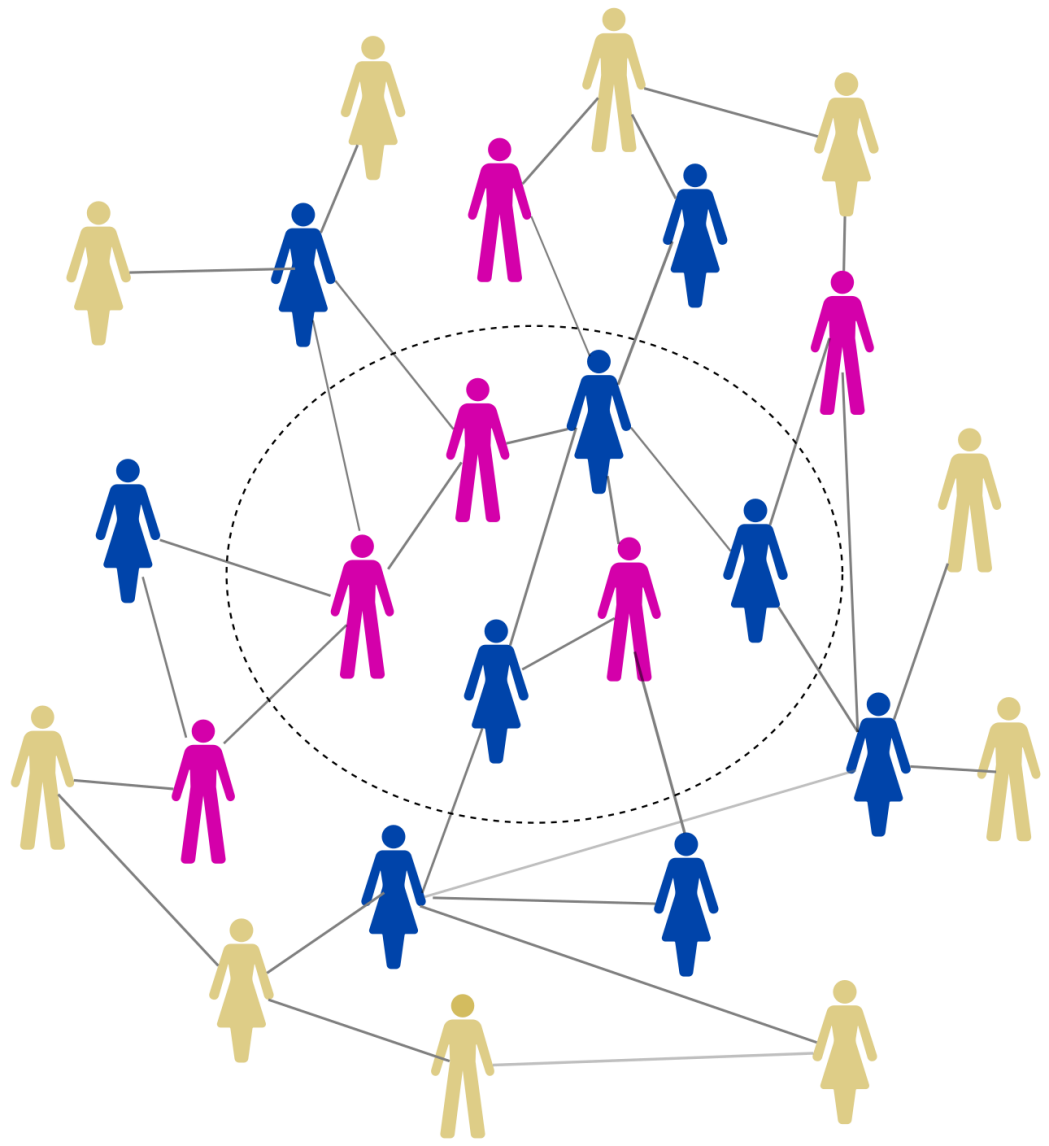
An illustration of the Sixhumene study sampling methodology. Stage 1 respondents are those within the dotted circle; Stage 2 respondents are contacts of Stage 1 respondents, those in darker colour outside the circle. Both groups will be interviewed. Contacts of Stage 2 respondents, those in paler colour, will not be interviewed.

Sixhumene will be an open cohort study where follow-up of each participant is planned to be 32 months from their enrolment date. The study sample will be recruited in two stages, using a one-step snowball approach as demonstrated in Figure 1. The initial sample (Stage 1) will be of everyone within the dotted circle; they will then be asked to refer into the study their close social contacts (those directly connected to them in the figure) who will form the sample at Stage 2. The study will be centred on three geographically defined communities purposively selected to have maximum variation in expected social dynamics. Specifically, one will be in a rural area with low population density spread across a wide area, one will be urban (in KwaMsane) and the third will be in a small village with well-defined borders on three sides.

Stage 1 participants will be identified from the most recent AHRI census (currently conducted on a rolling basis reaching each household once every four months) of the three study areas. All census respondents are asked if they consent to being re-contacted based on the information they provide, and if their data can be linked across AHRI studies and to other government data (e.g., clinical information). For each community, we will make a listing of the 250 resident individuals aged 16–24 years living closest to a GPS point chosen to be central within the community (e.g,, a school, a small shop, a church), with an expectation based on previous AHRI studies of an 80% contact and consent rate ( Mthiyane *et al*., 2021; Rochat *et al*., 2015). To account for some households not having been contacted during the coronavirus disease 2019 (COVID-19) pandemic – which necessitated a shift to largely telephonic surveillance ( Siedner *et al*., 2020) – we will also visit any households not contacted since January 2020 that lie within the maximum distance of any household containing the 250 listed individuals. Any eligible individuals living in these uncontacted households will be additionally invited to participate. If our enrolment target of 200 participants per area is not reached, we will then expand outwards from the initial area based on GPS-location of age-eligible residents’ households to add individuals until we reach 200 enrolled persons.

Each potential participant will be approached at their listed home location by a trained AHRI research assistant. After agreeing that they meet the inclusion/exclusion criteria they will be asked to provide informed consent. Informed consent will be requested based on a discussion of the study aims and procedures between the research assistant and the potential participant. Potential participants will have the opportunity to review the consent form and ask questions prior to signing. It will be clearly stated to potential participants that they may withdraw from the study at any time during the study and that their access to any form of assistance or care will not be adversely affected if they decline to participate in this study. All discussion will be conducted in isiZulu or English, based on the preference of the potential participant; consent forms will be available in both languages. Informed consent and assent (for those age <18 years) will be documented electronically on a study tablet computer including an electronic signature. Participants will be provided with a paper copy of the information sheet and informed consent document.

Since this study will potentially enrol minors (aged under 18 years), specific consent procedures will exist for this group. Anyone aged under 18 years at study enrolment will not be spoken to until a parent or guardian has provided informed consent. The process will be the same as that for potential participants, but consent will be sought for their child/ward to participate. If such consent is provided, we will ask the minor to provide informed assent to participate. This assent will follow the same process as for adults, but will be an assent rather than consent. If either the parent/guardian or the minor declines to provide consent, the minor will not participate in this study.

In line with existing AHRI standard operating procedures, if the individual is not present, the research assistant will request contact details from household members or neighbours and will then make contact and arrange a time and location to meet – either at the potential participant’s home or another convenient location where privacy can be maintained.

We will ask these Stage 1 participants to name key social contacts who provide advice, support (emotional, informational, financial, social) or are otherwise important to them. Based on pilot work ( Harling *et al*., 2018), we expect them to name (aside from one-another) ~350 unique peers (individuals aged 16–24) and ~550 unique older adults, for a total sample size of ~1500 – although many named others will be mentioned by multiple respondents. In our pilot the interquartile range of the number of people named by each respondent was 2–4, with a maximum of eight. All those identified by a Stage 1 participant who are resident in the AHRI PIP area will be invited to participate in the study as Stage 2 participants. We will first provide a uniquely identified recruitment coupon to the Stage 1 participant to pass on, asking their contact to call the study team. If such contact is made, the Stage 1 participant will receive R10 (~$0.5) in phone credit for each contact made up to a maximum of six contacts (limited to avoid inducing spurious named contacts). If no contact is made within 7 days, with the stage 1 participant's prior permission we will try to contact the Stage 2 potential participant directly, using either contact information provided by the Stage 1 participant, or other prior AHRI information (provided consent has previously been given for this re-contact). We discuss the ethics of this use of personal identifiable information below in the Social Network Identification subsection of the Ethical Considerations section.

The eligibility criteria for Sixhumene participation are:

- Aged 16–24 years at enrolment (Stage 1 participants) or aged 16 years or older (Stage 2 participants);
- Resident within the AHRI PIP area at enrolment;
- Be willing and able to consent to all study procedures (if aged 18 or older) or be willing and able to assent and have a parent or guardian willing and able to consent (if aged 16 or 17);
- Be able to demonstrate comprehension of the study objective and procedures, based on a conversation with the field staff member.

Staff are also able to exclude anyone where they have a clear reason to believe that inclusion would jeopardize the health or well-being of the participant or staff member, or would interfere with study conduct.

This study will be an open cohort based on the social contacts of the 600 Stage 1 participants. Post-baseline enrolment as a Stage 2 participant will be offered to any otherwise study-eligible individual who at any visit other than the last one:

- Is named as a new social contact by a Stage 1 participants;
- Has turned age 16 while living within a household that was study-eligible for Stage 1 at baseline; or
- Has migrated into a household that was study-eligible for Stage 1 at baseline and is aged 16-24 years.

All participants will be offered R20 (~£1) phone credit for each interview, in recognition of the time provided by the participant. This credit will be for any participation in the study visit, so will not be dependent on participation in any specific aspect of the study (e.g., providing a blood sample). This amount is in line with the current South African recommendations ( National Health Research Ethics Committee, 2012; South African Health Products Regulatory Authority, 2018).

### Study visits and procedures

**Figure 2.**
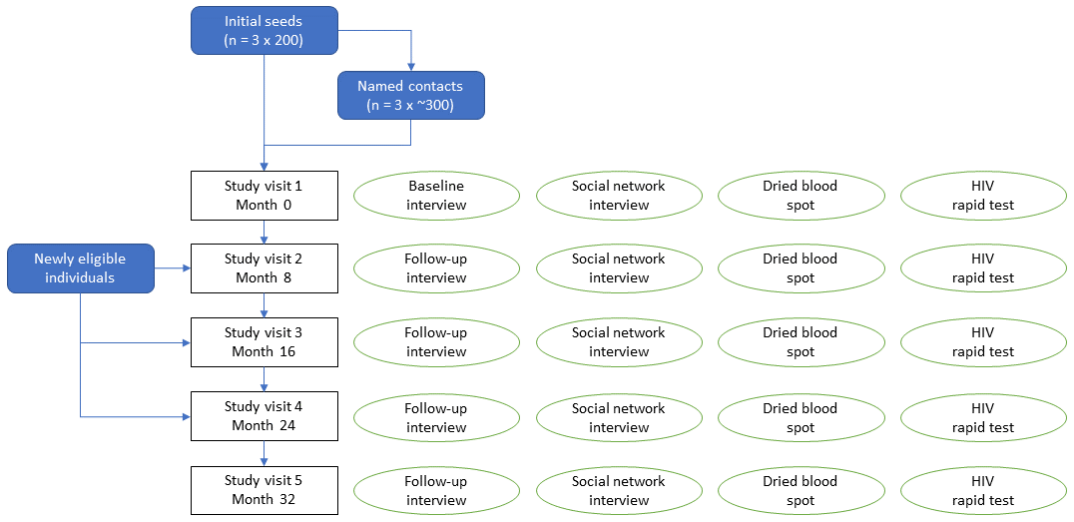
Sixhumene study schedule.

The study aims to interview all respondents at baseline, and then at 8, 16, 24 and 32 months post-enrolment ( Figure 2). Once informed consent has been provided, procedures at Visit 1 will begin with a tablet computer-based questionnaire that contains both interviewer-led and self-interview material. The questionnaire will use REDCap (Richmond, VA; Vanderbilt University) and Network Canvas ( Birkett *et al*., 2021) software for data collection. In addition to the questionnaire, we will ask for a capillary blood sample via finger-prick from which five dried blood spots (DBS) will be created.

All questionnaire and blood sampling procedures will be repeated at each follow-up visit, although questionnaire modules will be shortened to capture only updates from previous visits. For Visits 2–4, if the participant is continuously away from the AHRI PIP surveillance area for more than two months, we will attempt to conduct a further-shortened interview (including some aspects of the social network interview) by telephone or in-person at their current location; the final study visit will only be conducted in-person to allow for DBS provision. For all follow-up visits, the study team will contact each participant in the 30 days prior to the planned visit date to schedule a time and place to conduct the study visit, with the aim of minimizing participant inconvenience and maximizing study efficiency. Participants are free to withdraw from the study at any point by contacting the study team; since this is not an intervention study, there are no specific withdrawal procedures, but participants will be offered the opportunity to have all their prior data removed from the study dataset. Study participation ends following Visit 5, death or at withdrawal from the study, whichever happens first.

***HIV point-of-care testing*.** All participants will be offered the opportunity to take a point-of-care rapid HIV test at each visit, including if they do not consent to provide a blood sample for research. Testing will be performed by Research Assistants trained in HIV Testing Services (HTS) in accordance with national policy ( National Department of Health, 2015). The test will be done in a private space after written informed consent. While largely following the South African national HIV testing services protocol, our protocol will involve two simultaneous rapid HIV tests with test kits of different formats from different manufacturers. Initially these are One Step HIV (1/2) (Guangzhou Wondfo Biotech Co. Ltd.) and ABON HIV 1/2/O Tri-line (ABON Biopharm (Hangzhou) Co. Ltd) rapid tests. If results from the two tests are concordant for HIV-1 status, the participant will be informed accordingly. If they are discordant, an appointment will be made for a nurse to visit the individual at home or at a mobile clinic to perform phlebotomy to obtain a venous blood specimen for laboratory enzyme-linked immunosorbent assay (ELISA). Participants who test concordant positive for HIV and who have either not previously been diagnosed, or have been previously diagnosed but have not engaged with care, will receive a referral form for attendance at the public-sector clinic of their choice within 7-10 days. With participants’ consent, those receiving a referral but not linked to care within two weeks will receive a single Short Message Service (SMS) reminder and if they have still not linked within a month a trained nurse will follow-up by telephone.

***Data linkage*.** In addition to continuously collecting information on residents in the PIP surveillance area since 2000, AHRI has memoranda of understanding from government departments allowing access to service delivery data (from public health, welfare, home affairs, and education authorities). Many residents have previously provided consent for these data to be linked to AHRI-collected information. We will ask participants in the current study for permission to link their data from this study to other AHRI-linked data. This will lower the burden to participants by reducing the collection of duplicate data by different studies, and increases the reach of the current study, e.g., by allowing assessment of clinical outcomes such as linkage-to-HIV-care. All these datasets are only linked by AHRI’s unique pseudonymization key.

### Study instruments 

The baseline and follow-up interview ( Figure 2) will consist of an interviewer-led REDCap questionnaire to collect social and health information about the respondent. This will include: 1) socioeconomic – educational, employment and residential circumstances; 2) healthcare – service utilization (particularly relating to sexual and reproductive health (SRH)), HIV history (testing, treatment and prevention); 3) sexual health – knowledge, sources of advice, beliefs, descriptive and injunctive norms; 4) sexual behaviour, including contraceptive use; 5) substance use – tobacco, alcohol, marijuana; 6) psychosocial factors – HIV-related stigma ( Kalichman *et al*., 2005), common mental disorders ( Patel *et al*., 1997), hope ( Abler *et al*., 2017), social support ( Broadhead *et al*., 1988; Epino *et al*., 2012); and 7) experiences of COVID-19. The questionnaire emphasizes collecting information about knowledge, attitudes and behaviours expected to be correlated within families and communities.

A separate social network interview will be conducted using Network Canvas, comprising three components. First, name generators will be used to elicit the names (or nicknames) of people important to the respondent. We will use five role-specific name generators to capture individuals who provide emotional, informational, financial, physical and socialization support, plus any sexual partners in the past 12 months and any ‘important others’ not otherwise named. Follow-up name interpreter questions will be used to gather details about the contact, about the relationship between the respondent and contact, and the relationships between contacts. The baseline versions 1.4 of both interviews are contained in the *Extended Data* documentation ( Harling, 2022).

Central to the study design, all respondents (stage 1 and stage 2) will be asked to help identify their contacts in the wider social community, to allow the construction of a sociocentric dataset. This name identification process will begin by asking for key details about each contact: first name, last name, telephone number and community of residence (isigodi). These details will be used to conduct a fuzzy logic search of a minimal AHRI census dataset held on the encrypted tablet computer and return a shortlist of potential matches. To confirm the contact’s identity (where found), the respondent will be asked to confirm secondary information (birthday/age, parents’ names). All personal identifiers will be stored securely on AHRI servers and used to conduct probabilistic linkage both within the Sixhumene study database and using other AHRI-held datasets. Initial linkages identified using distance-based measures of similarity (e.g., Jaro-Winkler ( Gomaa & Fahmy, 2013)) will be validated by study team members to maximize plausibility. The two matching approaches (respondent-led and probabilistic) will be compared to confirm identities of stage 2 participants to assess validity. We discuss the ethics of using personal identifiable information below in the Social Network Identification subsection of the Ethical Considerations section.

All study materials were developed in English, translated and back-translated into isiZulu, and then tested both within the study team and through field-based piloting.

### Data management

***Interview data*.** Interview responses for this study will be captured on tablet computers during in-person or telephonic interviews. Tablet data will be transferred to database servers within the AHRI secure cluster server located within AHRI’s Somkhele site daily across a secure internal Wi-Fi system. Both the tablets and AHRI’s servers (which are regularly backed-up) are encrypted at rest (i.e., while not being transferred) and password-protected. Tablets will be charged overnight in a locked cupboard within access-controlled rooms on the access-controlled AHRI Somkhele site; any paper recruitment lists or activity logs will be secured within a similar locked cupboard. Server access control is managed through Microsoft Active Directory with minimum password complexity and compulsory password change policies. AHRI also uses industry-standard malware and intrusion detection with at least annual penetration tests by a reputable outside security audit company. All servers use a two-tier uninterruptable power supply comprising switch-over to a local diesel-powered generator and controlled shutdown.

During the social network module respondents will be asked to identify their close personal contacts based on personally identifiable information using a dataset within Network Canvas. This dataset will be uploaded to the mobile devices from the AHRI secure server within the AHRI Somkhele building and will contain only identifiers (names, residential location, date of birth, family member names, telephone numbers) – no information on their lives such as social, demographic or health information will be present. Interviewers will not be able to access the dataset, only to query it using search terms.

***Dried blood spots*.** DBS sample cards will be pseudonymized from the point of collection, being identified only by a barcode captured on the tablet computer. The cards will be returned to AHRI Somkhele daily and stored at the on-site laboratory until they are ready for transfer to AHRI Durban (2.5 hour drive away), a trip made every work day. At AHRI Durban DBS cards will be processed for HIV and HSV-2 testing within an access-controlled laboratory; unused DBS will be stored in freezers in access-controlled rooms for potential future research which would require additional approval from research ethics committees. Results from HSV-2 and HIV tests will be entered into a REDCap form on AHRI computers and then saved on the AHRI server.

Data transfer from AHRI’s servers will only occur post-pseudonymization. The pseudonymization key, which allows data from multiple studies at AHRI to potentially be linked together is held by AHRI’s Chief Information Officer and the head of Research Data Management (RDM). In line with AHRI’s privacy policy, the key will never be provided to study team members outside RDM. Pseudonymized data transfer will be conducted via AHRI’s password-protected online data repository or password-protected download links. Following study completion and assessment for disclosure risk using sdcMicro ( Templ, 2008), the pseudonymized dataset will be given a Digital Object Identifier and made available for download via SSL encrypted links by researchers after signing a Data Use Agreement with AHRI. Pseudonymized analytic datasets will be stored by the study team on password-protected servers or password-protected and encrypted personal computers with automatic screen lock-out. Data will be maintained at AHRI indefinitely in line with their standard policies.

Parental notification of HIV test results is not required by law in South Africa. All HIV and HSV-2 test results will be kept confidential, and since HIV point-of-care testing will be offered, results of tests using DBS will not be returned to participants. HSV-2 treatment is symptom management; any participant describing symptoms will be referred to a local clinic.

### Material storage and laboratory procedures

We will use Whatman 903 filter cards to collect DBS samples. The blood specimen will be collected using single-use automatically retracting safety lancet needles after cleaning the injection site with an alcohol swab. The DBS will be dried by waving the filter paper, placed into a barcode-labelled envelope within a larger zip-lock bag and transported in a cooler bag. Specimens will be submitted daily at AHRI Somkhele, held overnight at 2-8 °C at Somkhele Laboratory and then transported to the AHRI Diagnostic Research Laboratories in Durban on the next working day. At the laboratory DBS cards will be stored at -20°C for a period of 20 years from study end, or until depleted. 

***HIV testing from DBS*.** HIV testing will be conducted using the Genscreen Ultra HIV 1/2 Ab-Ag assay (Bio-Rad) on DBS samples ( Solomon *et al*., 2002). A 4.7mm diameter punch of a DBS spot from each card will be made using a DBS puncher (Wallac) and incubated overnight in 200ul of calcium and magnesium free Phosphate Buffered Saline (PBS) for a maximum of 16 hours at 4°C. The assay will then be performed with 200ul of the eluent in accordance with the manufacturer’s instructions. The mean cut-off values will be multiplied by a factor of 2.0 before determining the index value for each sample. The results are reported as positive (index value >1.10), equivocal (index value of ≥ 0.90 but ≤1.10) or negative (index value <0.90). All initial equivocal results will be re-tested and those that re-test equivocal will be reported as such.

***HSV-2 testing from DBS*.** HSV-2 testing will be conducted using the HerpeSelect® 2 ELISA IgG assay (FOCUS Diagnostics, Cypress, California, USA). The assay has been validated for use with serum samples in South African settings ( Delany-Moretlwe *et al*., 2010; Mujugira *et al*., 2011), and use with DBS samples has been validated against serum in India ( Schneider *et al*., 2010). The assay has also been optimised for use with DBS in the AHRI Diagnostic Research Laboratory following comparative testing with plasma samples (including select testing of some plasma samples by an external accredited pathology laboratory). A 6mm diameter punch of a DBS spot from each card will be made using a DBS puncher (Wallac) and incubated overnight in 150ul of Assay Diluent for a maximum of 15 hours at 4°C. The assay will then be performed with 50ul of the eluent in accordance with the manufacturer’s instructions. The mean cut-off calibrator absorbance values will be multiplied by a factor of 1.5 before determining the index value for each sample. The HerpeSelect ® 2 ELISA IgG results are reported as positive (index value >1.10), equivocal (index value of ≥ 0.90 but ≤1.10) or negative (index value <0.90). All initial equivocal results will be re-tested and those that re-test equivocal will be reported as such.

### Analytic strategy

***Outcome measures*.** Our primary outcome measures will be biologically measured HSV-2 and HIV incidence, defined as a positive test result following a prior negative one. We will additionally measure linkage to public sector HIV treatment through linked data collected in local clinics, defined as a visit to one of the 11 local clinics within 60 days of a positive point-of-care HIV test. Additionally, we will consider as secondary outcomes several self-reported behaviours and attitudes that may serve to mediate these health outcomes. Health behaviours will include sexual intercourse (ever and age of debut), lifetime number of partners, numbers of children and contraceptive use (type and frequency). SRH attitudes will include beliefs regarding perceived effectiveness of, and respondents’ ability to use, four key HIV prevention methods (abstinence; faithfulness; condoms; knowing partner).

***Key social network measures*.** We will summarize egocentric social contact quantity both as counts of named individuals and based on the frequency of contact or support provision reported summed across all contacts ( Harling *et al*., 2020). These values will also be subdivided into kin and non-kin. We will additionally consider contact results for the outcomes above (e.g., HIV and HSV-2 seroprevalence and behavioural prevalence amongst named contacts). Finally, we will consider the injunctive & descriptive norms reported by respondents about preventing pregnancy (long-acting reversible contraceptives for females; condom use for males), HIV testing and antiretroviral therapy use – where injunctive norms are what respondents believe their social referents think the respondent should do, and descriptive norms are what respondents believe these referents do themselves ( Cislaghi & Shakya, 2018).

***Data analysis*.** Stage 1 participants will be drawn in a random manner and thus broadly represent the study population; however, those at Stage 2 will by design not be representative so we will calculate sampling weights for any analysis aiming to produce population prevalence. We will also build non-response weights for Stage 1 sampled individuals and for follow-up.

We will first describe baseline cohort characteristics using univariate and bivariate statistics with parametric or non-parametric tests of difference by gender and age, depending on the distributions of the data. For all associational analyses we will use models that explicitly allow for network-structure dependence while also adjusting for respondents’ non-network characteristics.

For cross-sectional analysis of baseline associations between outcomes and contact’s characteristics allowing for network structure, we will use Autologistic Actor Attribute Models (ALAAMs) which evaluate diffusion across a static network building from the Exponential Random Graph Model framework ( Daraganova & Robins, 2013; Parker *et al*., 2021). For longitudinal analysis we will use Stochastic Actor-Orientated Models (SAOMs), which are Markov-chain dynamic simulation models that jointly evaluate changes in network structure and outcomes, allowing us to explicitly separate social selection and social influence, and ensure temporal ordering of effects ( Steglich *et al*., 2010). We will use multiple imputation ( Wang *et al*., 2016) and model-based procedures ( Huisman & Steglich, 2008) to account for missing individuals and missing ties between individuals.

*Primary analyses*. To evaluate whether HIV and HSV-2 outcomes are predicted by the outcomes of their social contacts, we will use a SAOM to evaluate whether having more social contacts living with HSV-2 or HIV, or unlinked to care is associated with higher hazard of HSV-2 seroconversion, HIV seroconversion and not linking to care once aware of positive HIV status. To evaluate whether peer contacts raise the risk of such outcomes, we will additionally use as exposures the proportion of contacts who are peer-aged and the ratio of peers to family members – based on numbers named and frequency of support provided.

*Secondary analyses*. First, to evaluate how social processes are associated with risk behaviours, we will use SAOMs to evaluate whether: (i) changes self-reported numbers of partners and numbers of condomless partners are positively associated with the values reported by their peer contacts; or (ii) individuals become friends with peers who report the same level of sexual behaviour as they do. Second, to identify who is at greatest risk of acquiring HSV-2 and HIV we will use ALAAMs to conduct exploratory, predictive modelling to measure how incident HSV-2 and HIV acquisition is predicted by characteristics of: (i) individuals; (ii) their social contacts; and (iii) their immediate social network structure. Third, we will identify influential individuals comparing several approaches, including: i) local (degree) and community and global (betweenness) centrality; ii) social status algorithms based on non-reciprocal ties ( Ball & Newman, 2013); iii) respondent nominations of influential others, using both global and community maxima ( Starkey *et al*., 2009); and iv) Key Player-Positive algorithms to identify the most efficient set of network diffusers ( Borgatti, 2006). For each method we will use regression analysis to evaluate which characteristics are associated with being influential.

***Sample size calculation*.** The number of planned participants was based on a power calculation for our primary objective relating to HIV incidence. Complete follow-up on 1000 15–29 year olds would provide 2500 person-years at risk. Assuming baseline HIV prevalence of 15%, and 20% of person-time lost to follow-up, we will have 1700 person-years of at-risk follow-up. If HIV incidence in this cohort is at least 4% per annum in this age-group, which was the case at the time of study design ( Vandormael *et al*., 2019), we would have 80% power to see a hazard ratio of 2:1 for any 50:50 split of the cohort (e.g. those with more/fewer HIV-positive social contacts or those with more/fewer peer social contacts). The even higher HSV-2 incidence in this age-cohort (16.5% per annum for 13–22 year old females ( Fong *et al*., 2021)), means that even if male incidence is half that of females, we should have 86% power to a hazard ratio of 1.5:1.

### Ethical considerations

This study has been reviewed and approved by the University of KwaZulu-Natal’s Biomedical Research Ethics Committee (0359/2019) and University College London Research Ethics Committee (15231/009), and by the KwaZulu-Natal Department of Health (KZ_201911_012). The design of the study was discussed with AHRI’s Community Advisory Board (CAB) prior to finalization, and their formal approval gained as part of the ethical review process. We have designed the consent procedures to provide clear guidance on what the study entails, both in terms of participants’ contributions and the information they will be providing on their social contacts. Nevertheless, there are very important ethical issues to consider, given the combination of the social network methodology and the highly sensitive subject-matter of the study.

***Psychological harm*.** Both questionnaire topics and HIV testing may cause psychological distress to respondents. Study staff will be trained in minimize participant discomfort, however, if a respondent shows distress, they will be reminded that they can pause or withdraw from the study at any time. The study information sheet includes contact details for discussion of any study-related problems (free of charge via the ‘Please Call Me’ facility). Referral to local clinics. or in severe cases to local hospitals, will be made based on an assessment by clinical staff.

***Social protection*.** Study questions may reveal actions on the part of participants or their social contacts that break South African law. In particular, it is illegal for anyone aged 16 or over to engage in a sexual act with someone aged under 16 years where the age difference is more than two years, even with consent of the younger person ( Bhamjee *et al*., 2016). Since we will be asking participants whether they have had sex with social contacts, and we will be identifying social contacts and will know both ages, we may find that we have captured a report of an illegal activity. South African law requires such knowledge to be reported to the relevant authorities.

However, age-disparate relationships between teenage girls and in their twenties or older are normative in South Africa – one third of 15–19 year old females report a recent sexual partner four or more years older than them ( Shisana *et al*., 2014). Reporting such relationships may cause psychological and social harm to the younger person, potentially requiring them to incriminate their partner in court ( Bhamjee *et al*., 2016). It is also likely to create mistrust of clinical services and implementing partners in the community, resulting in reduced future disclosure, potentially worsening population health.

Given the conflict thus generated between researchers’ legal responsibility to report and their ethical responsibility to maintain confidentiality ( Strode & Slack, 2009), we will follow a previously suggested approach ( Strode & Slack, 2014). Specifically reports of sexual activity with a 12–15 year-old individual by study participants where the age difference is greater than two years are acknowledged to be illegal, but will not be reported unless that sexual activity is clearly exploitative. This exploitation will be considered by a multidisciplinary team, including AHRI’s dedicated Participant Protection Lead, with the goal of assessing whether the adolescent’s ability to consent to the sexual activity has been compromised by factors such as coercion, violence or a lack of power. More generally, if any field staff member becomes aware of potential child protection issues (involving participants or others) during their work, they have been trained to ask relevant questions and report the situation to their manager for discussion with the Participant Protection Lead. In all cases, the primary AHRI child protection standard operating procedure (SOP) will be followed, including referral and notification of authorities where appropriate.

***HIV status disclosure*.** HIV serostatus disclosure has the potential to cause substantial harm. We have therefore implemented multiple measures throughout the study to minimize this risk, in line with AHRI standard operating procedures. Several steps will be taken during interpersonal interactions. First, all interviews and HIV tests will be conducted in locations from which discussions cannot be overheard by others; if such a location is unavailable study staff will be instructed not to conduct interviews. Second, participant names will not be included on any documentation that contains a result. Third, HIV serostatus at baseline is not an exclusion criterion, to avoid deductive status disclosure to friends and family. Fourth, parents/guardians will be told that HIV and HSV-2 test results are confidential and that the study team cannot provide test results to them. Fifth, on request, study staff will conduct a finger prick without taking blood if the respondent is concerned about stigma associated with not having a finger prick (e.g., if they had had HIV infection documented at a prior study visit). Additionally, there are multiple data protection measures implemented post-interview, including the use of password protected and encrypted tablet computers, pseudonymized data storage and separation of identities from all other study information and strict data access controls.

***Social network identification*.** The provision of third-party identities is central to the goals of this study. However, in naming social contacts and describing their relationships respondents may provide private information, including relationship characteristics (e.g., sexual behaviours, level of trust) or even the relationship’s existence, which might cause harm if passed to others. Furthermore, while asking respondents for third-party information is common (e.g., when conducting proxy interviews with household heads, or asking about sexual behaviour with spouses), such questions mean some individuals are included in the study data without providing consent. We consider the information being provided about social contacts as being the respondents’ perceptions of their relationships with others, and thus theirs to provide if they wish ( Klovdahl, 2005). Nevertheless, this does not negate the potential for harm that could arise if study information were made public.

For third-party identities specifically, we follow an existing ethical framework that recommends collecting such information only when three criteria are met ( Lounsbury *et al*., 2007):

1. *No other method is available*. This is the case for any sociocentric analysis, since only by asking respondents to identify their social contacts can connections between responses be made ( Klovdahl, 2005);

2. *Third-party identities are maximally protected*. This study will never collect third-party identities outside of an encrypted electronic environment. These identities will be held within datasets using AHRI-specific identifiers, the linkage for which is held by key employees only. At no point will information provided by one participant be provided to another;

3. *Benefits outweigh harms*. Stakeholders here include respondents, third-parties and the broader public. Respondents’ social connections are central to understanding social norms, which in turn are central to understanding HIV-related risk in a community with a generalized HIV epidemic. In this setting, these data therefore have a strong potential to benefit all community members. To minimize potential harms, we will intentionally not focus on sexual relationships, asking only sufficient questions to identify potential transmission pathways, rather than collecting detailed sexual behaviour information. 

### Staff safety

Any field-based activities potentially generate risks. We will take various steps to minimize these risks, in line with AHRI standard operating procedures. These include:

1. Transport to participant homes will be in regularly-serviced AHRI fleet vehicles; drivers have to pass an externally conducted driving competence test;
2. All field-based staff locations will be logged daily in a central register;
3. AHRI’s mobile Security Response Team will conduct regular drive-by checks with research assistants;
4. The study coordinator will review daily AHRI community security briefings;
5. AHRI’s on-site occupational nurse will be available to all team members, as well referrals to external counselling services;
6. A field team WhatsApp group will be overseen by the study coordinator to allow for real-time reporting of problems and call for backup;
7. The study coordinator will conduct regular debriefings of field teams to discuss past and potential future concerns.

### Public involvement

Members of the public will be involved in this study at several stages. First, the study proposal was presented to the AHRI CAB, which is drawn from the PIP surveillance area resident community to advise on all research conducted at AHRI. The AHRI CAB had previously stated to the PI the importance of understanding how HIV is spread among young people in the local area. Second, the study will be presented in each study community via a ‘roadshow’ or equivalent event held in a local outdoor public space. These roadshows provide an opportunity for AHRI staff, led by the Public Engagement team, to present the aims, planned activities and findings of the study to community members. Sixhumene will present both prior to study start and after study completion. Local community leaders – both traditional and elected – will be alerted to the study prior to commencement too. Finally, preliminary results will be presented to AHRI CAB for their feedback, comments and advice, which may lead to additional analyses or adjustments to how results are understood and presented. 

### Dissemination plan

The results of this study will be shared with AHRI’s CAB, and summarized in a format available to the local community through mediums such as radio presentations, leaflets and community meetings. The results will also be submitted to academic conferences and peer-reviewed journals for publication. Any manuscripts arising from this study will be published as open access articles in line with Wellcome Trust policy and Plan S. All manuscripts and presentations will be shared with the KwaZulu-Natal provincial Department of Health in advance of submission, in line with AHRI’s Memorandum of Understanding, and where of clinical or programmatic interest government staff will be engaged directly.

***Future data availability*.** The data generated in this study will be made available to the research community subject to careful controls. A standard dataset will be generated that includes all data collected this study except for personally identifiable information questions (e.g. name, location, phone number). This dataset will be linkable to other AHRI datasets and would be made available through the AHRI data repository. A second dataset will be generated that includes unique contact identifiers from the study. This data will be highly identifiable and since it contains sexual behaviour and HIV/HSV-2 status, has considerable potential to cause harm if made available to someone with malicious intent. Nevertheless, such data would provide an almost-unique insight into the social dynamics of a population, providing network structure and thus allowing analysis of community-level spread of ideas, behaviours and infections. Use of this dataset by non-study personnel would require a discussion with the study PI and a responsible AHRI officer, signature of an extended data use agreement and use of the data only through AHRI’s Virtual Data Enclave (VDE) run on the AHRI server and accessed through a Virtual Private Network (VPN). The VDE is isolated from both the user’s physical desktop and external access – so that researcher cannot accidentally or intentionally remove files. Removal of processed datasets or analysis results from the VDE will require AHRI Research Data Management approval to ensure no identifiable data is disclosed.

***Study status*.** The study commenced baseline recruitment on February 8 ^th^, 2022 at the first of three study areas. This process remains ongoing as of the end of April 2022.

## Discussion

Despite sexual health being inherently dependent the attitudes and actions of others, much research on how behaviour change spreads through communities and society does not explicitly measure how individuals are connected, how they influence one-another and how this translates into health behaviour and positive or negative sexual health outcomes. By building a socially connected cohort study that is constructed to maximize the chances of capturing entire social networks, and conducting regular interviews over three years, the Sixhumene study aims to examine all of these topics in a setting with high levels of sexually transmitted infections ( Francis *et al*., 2018; Vandormael *et al*., 2019) and unplanned pregnancies ( Rochat *et al*., 2013).

The data collected in Sixhumene should be directly useful in designing interventions to reduce poor sexual health outcomes in this and similar settings. Such interventions may involve changing who leads behaviour change efforts within communities – by targeting key influential individuals within groups (e.g., peers) or shifting which groups are targeted (e.g., from peers to siblings and cousins). Interventions could also adjust what messages to provide, e.g., from educational messaging to normative feedback, i.e., correcting misconceptions of what is normal among peers ( Chernoff & Davison, 2005). A key aspect of Sixhumene will be its ability to link individuals’ position within broad social networks (and associated risk for poor health outcomes) with personal characteristics. This is vital since observing full-community social networks requires substantial resource outlays, which often make them prohibitively expensive. Past work has shown that targeting individuals for interventions based on proxy measures of network position can in theory achieve much of the benefit that can be attained by using full-network information ( Yang *et al*., 2019); determining which measures are the best proxies is an important next step.

In addition to informing interventions directly, Sixhumene should provide important empirical network dynamics data that can both provide parameter estimates and assist with calibration and validation of mathematical modelling of infectious processes – both relating to sexual health and more broadly. For example, Sixhumene could inform questions relating to social support and conflict, educational homophily, uptake of innovations (e.g., PrEP, Covid-19 vaccines) and mental health. Our focus on making data available as widely as safety and privacy concerns allow should aid with all this work.

Last, but not least, the Sixhumene study has the potential to provide participants with insight into their own social lives. Preliminary presentations of the approaches through piloting and discussion with the community advisory board suggest that participants are able to evaluate their social world through the use of sociograms, and that this visualization process increases awareness of who they turn to for help and companionship. In addition, community-level social network maps can (if presented with care) create opportunities for discussion with members about the current and desired form of their social lives, opening the door to co-creation of community-relevant interventions ( Hinchliffe *et al*., 2018).

## Data availability

### Underlying data

No data are associated with this article.

### Extended data

Zenodo: Protocol: Mapping social networks, social influence and sexual health among youth in rural KwaZulu-Natal: the Sixhumene cohort study – Supplementary Material. https://doi.org/10.5281/zenodo.6323120 ( Harling, 2022).

This project contains the following extended data:

1. Supplementary Material 1.docx (Sixhumene study questionnaire)
2. Supplementary Material 2.docx (Sixhumene study information sheet and consent forms)

Data are available under the terms of the Creative Commons Attribution 4.0 International license (CC-BY 4.0).
