# Supplementary material for: Protocol: Mapping social networks, social influence and sexual health among youth in rural KwaZulu-Natal, the Sixhumene cohort study
Source: Wellcome Open Res. 2022 May 26;7:164. [Version 1] doi: 10.12688/wellcomeopenres.17896.1 (PMC9608251; doi:10.12688/wellcomeopenres.17896.1)
Supplement: Peer review history

# Peer review: Reviewer response for version 1

**DOI:** 10.21956/wellcomeopenres.19829.r50819

**Published:** 2022-06-27

**Authors:** Thespina Yamanis

**Affiliations:** 1 School of International Service, American University, Washington, USA

Thank you for inviting me to openly review the published article “Protocol: Mapping social networks, social influence and sexual health among youth in rural KwaZulu-Natal, the Sixhumene cohort study.”  This is a well-designed study with interesting research questions and hypotheses. The protocol paper included sufficient detail on the study design and methods. The proposed research will likely improve understanding about salient social network influences on sexual health in these communities.

 I have two clarifying questions. One question is regarding the sociocentric network data collection and the proposed analysis plan for these networks, which includes Stochastic Actor-Oriented Models (SAOMs). SAOMs require networks to be relatively stable over time. The methodology you will use to identify network contacts at each wave (all respondents will be asked to identify their contacts in the wider social community) may be vulnerable to missing data and nonresponse, especially if respondents consider the name identification process to be burdensome (e.g. the fewer contacts named, the shorter the time spent on the survey).  I wonder if perhaps you might limit the sociocentric network data collected to particular sociocentric networks of interest (e.g. close peers or friends) in order to minimize the network data collected; this strategy may result in collecting data that are more usable for SAOMs.

 Another clarifying question is regarding the collection of sexual network data. In the Ethical Considerations section on page 10, the authors state that they will intentionally not collect data on sexual relationships in order to minimize potential harms. However, in the description of the network data to be collected, it explicitly states that the authors will use name generators to ask about any sexual partners in the past  12  months  (top of page 7).  It is unclear to what extent the authors are “not collecting data on sexual relationships” – please clarify.

 Finally, a minor point is that the first sentence of the discussion is missing the word “on”.

Is the study design appropriate for the research question?

Yes

Is the rationale for, and objectives of, the study clearly described?

Yes

Are sufficient details of the methods provided to allow replication by others?

Yes

Are the datasets clearly presented in a useable and accessible format?

Not applicable

Reviewer Expertise:

HIV risk behavior, youth, social networks, Tanzania

I confirm that I have read this submission and believe that I have an appropriate level of expertise to confirm that it is of an acceptable scientific standard.

# Peer review: Reviewer response for version 1

**DOI:** 10.21956/wellcomeopenres.19829.r50827

**Published:** 2022-06-13

**Authors:** Susan Cassels

**Affiliations:** 1 Department of Geography, University of California, Santa Barbara, Santa Barbara, USA

Thank you for the opportunity to openly review the published article “Protocol: Mapping social networks, social influence and sexual health among youth in rural KwaZulu-Natal, the Sixhumene cohort study.” This is a very thorough protocol paper, and the proposed research has potential to be very impactful. Data from this research can improve parametrization of mathematical models of HIV transmission dynamics, and identify the most salient peer influences on sexual behavior and health.

 The motivation of this study is very strong, and the authors outline their research aims and hypothesis well. I have one minor clarifying question: the authors propose two different mechanisms in which individuals have peers with similar behaviors. First, over time young people change their behaviors to mirror peers. Second, young people selectively form relationships with people similar to them. This is an age-old question in social network analysis literature. Do the authors think one mechanism might be more salient than the other? The data analysis section did not flush this out in detail: specifically, how these two mechanisms can be compared over time. (See this useful reference: Goodreau *et al*. 2009: “Birds of a feather, or friend of a friend? Using ERGMs to investigate adolescent social networks”.) ^1^

 The authors have already received IRB approval for this sensitive research. One of the trade-offs of conducting this sensitive research is that they are not asking young people to name sexual partnerships. Rather, they are using name generators to capture individuals who provide emotional, information, financial, physical, and socialization support. I am curious how significant this data limitation might be. I imagine that sexual partnerships are quite influential and informative in young people’s views on sexual health and behaviors. Are there other data that suggest an overlap in sexual partnerships and individuals who provide these other forms of support? Are they the same or different people?

 It appears that this research is relying heavily on Network Canvas. Readers of this protocol might be interested to learn more about this program. Is it publically available? Consider providing a link to this resource.

 There is a small typo on page 4, last paragraph (“will potentially enrol [enroll] minors”).

Is the study design appropriate for the research question?

Yes

Is the rationale for, and objectives of, the study clearly described?

Yes

Are sufficient details of the methods provided to allow replication by others?

Yes

Are the datasets clearly presented in a useable and accessible format?

Yes

Reviewer Expertise:

Demography, population health, HIV, population mobility, health geography

I confirm that I have read this submission and believe that I have an appropriate level of expertise to confirm that it is of an acceptable scientific standard.

## References

[1] : Birds of a feather, or friend of a friend? Using exponential random graph models to investigate adolescent social networks. *Demography* .2009;46(1) : 10.1353/dem.0.0045 103-25 10.1353/dem.0.0045 19348111PMC2831261
